# QPCR: Application for real-time PCR data management and analysis

**DOI:** 10.1186/1471-2105-10-268

**Published:** 2009-08-27

**Authors:** Stephan Pabinger, Gerhard G Thallinger, René Snajder, Heiko Eichhorn, Robert Rader, Zlatko Trajanoski

**Affiliations:** 1 Institute for Genomics and Bioinformatics, Graz University of Technology, Petersgasse 14, 8010 Graz, Austria; 2 Christian Doppler Laboratory for Genomics and Bioinformatics, Petersgasse 14, 8010 Graz, Austria; 3 Development Anti-Infectives Microbiology, Sandoz GmbH, Biochemiestrasse 10, 6250 Kundl, Austria

## Abstract

**Background:**

Since its introduction quantitative real-time polymerase chain reaction (qPCR) has become the standard method for quantification of gene expression. Its high sensitivity, large dynamic range, and accuracy led to the development of numerous applications with an increasing number of samples to be analyzed. Data analysis consists of a number of steps, which have to be carried out in several different applications. Currently, no single tool is available which incorporates storage, management, and multiple methods covering the complete analysis pipeline.

**Results:**

QPCR is a versatile web-based Java application that allows to store, manage, and analyze data from relative quantification qPCR experiments. It comprises a parser to import generated data from qPCR instruments and includes a variety of analysis methods to calculate cycle-threshold and amplification efficiency values. The analysis pipeline includes technical and biological replicate handling, incorporation of sample or gene specific efficiency, normalization using single or multiple reference genes, inter-run calibration, and fold change calculation. Moreover, the application supports assessment of error propagation throughout all analysis steps and allows conducting statistical tests on biological replicates. Results can be visualized in customizable charts and exported for further investigation.

**Conclusion:**

We have developed a web-based system designed to enhance and facilitate the analysis of qPCR experiments. It covers the complete analysis workflow combining parsing, analysis, and generation of charts into one single application. The system is freely available at

## Background

Amongst other high throughput techniques like DNA microarrays and mass spectrometry, qPCR has become important in many areas of basic and applied functional genomics research. Due to its high sequence-specificity, large dynamic range, and tremendous sensitivity it is one of the most widely used methods for quantification of gene expression. Moreover, due to the adoption of robotic pipetting stations and 384-well formats, laboratories generate a huge amount of qPCR data demanding a centralized storage, management, and analysis application.

Most software programs provided along with the qPCR instruments support only straightforward calculation of quantification cycle (Cq) values from the recorded fluorescence measurements. However, in order to get biological meaningful results these basic calculations need to undergo further analyses such as normalization, averaging, and statistical tests [1].

To this end, a variety of different methods have been published describing the normalization of Cq values. The simplest model (termed ΔΔ-Cq method) was developed by Livak and Schmittgen [2] which assumes perfect amplification efficiency by setting the base of the exponential function to 2 and uses only one reference gene for normalization. The model proposed by Pfaffl [3] considers PCR efficiency for both the gene of interest and a reference gene and is therefore an improvement over the classic ΔΔ-Cq method. Nevertheless, it still uses only one reference gene which may not be sufficient to obtain reliable results [4]. Hellemans *et al. *[5] proposed an advanced method which considers gene-specific amplification efficiencies and allows normalization of Cq values with multiple reference genes based on the method proposed by Vandesompele *et al. *[4]. It should be noted that these methods could differ substantially in their performance, because of the different assumptions they are based on.

Available software tools often cover only single steps in the analysis pipeline compelling researchers to use multiple tools for the analysis of qPCR experiments [5-8]. However, these tools do not share a common file format making it difficult to analyze the experimental data. Additionally, no standardization of methodology has been established that would be needed for relatable comparison between laboratories [9]. Recently, the Minimum Information for Publication of Quantitative Real-Time PCR Experiments (MIQE) guidelines [10] were published which are intended to describe the minimum information necessary for evaluating and comparing qPCR experiments. Based on a subset of these guidelines the XML-based Real-Time PCR Data Markup Language (RDML) [11] was proposed which tries to facilitate the exchange of qPCR data and related information between qPCR instruments, analysis software, journals, and public repositories. These efforts could allow a more reliable interpretation of qPCR results if they were accepted in the qPCR community.

The lack of complete or partial assessment of error propagation throughout the whole analysis pipeline may result in an underestimated final error and could therefore lead to incorrect conclusions. Moreover, the analysis of experiments using tools that make invalid biological assumptions can cause significantly wrong results as reported in [8].

To the best of our knowledge, there is no single tool available which integrates storage, management, and analysis of qPCR experiments. Hence a system enabling comparison of results and providing a standardized way of analyzing data would be of great benefit to the community. We have therefore developed QPCR, a web-based application which supports: a) technical and biological replicate handling, b) the analysis of qPCR experiments with an unlimited number of samples and genes, c) normalization using an arbitrary number of reference genes, d) inter-plate normalization using calibrators, e) assessment of significant gene deregulation between sample groups, f) generation of customizable charts, and g) a plug-in mechanism for easy integration of new analysis methods.

## Implementation

The QPCR system was implemented in Java, a platform independent and object-oriented programming language [12]. The application is based on the Java 2 Enterprise Edition (J2EE) three-tier architecture consisting of a presentation-, business -, and database-layer. A relational database (PostgreSQL or Oracle) is used as the persistence backend. The business layer consists of Enterprise Java Beans (EJB) and is deployed on a JBoss [13] application server. The presentation layer is based on the Model-View-Controller (MVC) framework Struts [14] and uses Java Servlets and Java Server Pages.

In order to enhance usability current web technologies have been extensively used in this application. AJAX functionality has been incorporated into the application using the open-source library DWR [15]. This technology allows asynchronous loading of data without the need to reload the page thus providing a desktop like application behavior. Multiple JavaScript libraries (Prototype [16], JQuery [17]) have been used that allow executing functions on the client side and therefore remarkably improve the usability of the application. Charts are generated using the open-source Java library JFreeChart [18] and all charts are created either in the lossless PNG format or as a scalable vector graphic (SVG).

All algorithms, calculation methods, and data file parsers used by the application are integrated through a plug-in mechanism which allows simple extension with additional qPCR data formats and analysis approaches. For each class that uses the plug-in mechanism a specific interface needs to be implemented in order to support another vendor or implement an additional analysis method. The new Java classes are then automatically detected by the QPCR application.

Currently the data file parsers support files generated by Applied Biosystems (ABI 7000, ABI 7500, ABI 7900) and Roche LightCycler (LightCycler 2.0, LightCycler 480) [19] systems as well as a generic file format based on comma separated values (CSV). Since not all fluorescence measurements can be extracted from data files created by the qPCR instrument systems, additional export files are required to parse all relevant data.

Analysis methods that calculate Cq and amplification efficiency values are computationally expensive and are therefore executed asynchronously and do not interfere with the QPCR web interface. They are designed to operate on a per well basis and report the current progress of the calculation. Normalization methods and statistical tests are not time consuming processes and are therefore executed in real time.

The QPCR application has been designed using the Unified Modeling Language (UML) [20]. The use of a UML representation improves maintainability as the application architecture is outright visible and provides an important part of the system documentation. We used the AndroMDA framework [21] to create basic EJB and presentation tier source code as well as configuration files based on the UML model. AndroMDA minimizes repetitive coding tasks, allows to easily extend or edit the architecture of the application, and helps maintaining the consistency between design and implementation.

The stored data is secured by a user management system which allows the definition of several fine grained user access levels and offers data sharing and concurrent access in a multi-centric environment [22]. Moreover, the application provides two configurations which assign the ownership of objects either to the submitter or to the submitter's institute. The latter setup provides the possibility to edit and analyze experiments by all users of an institute without the need to explicitly share objects.

## Results

**Figure 1:**
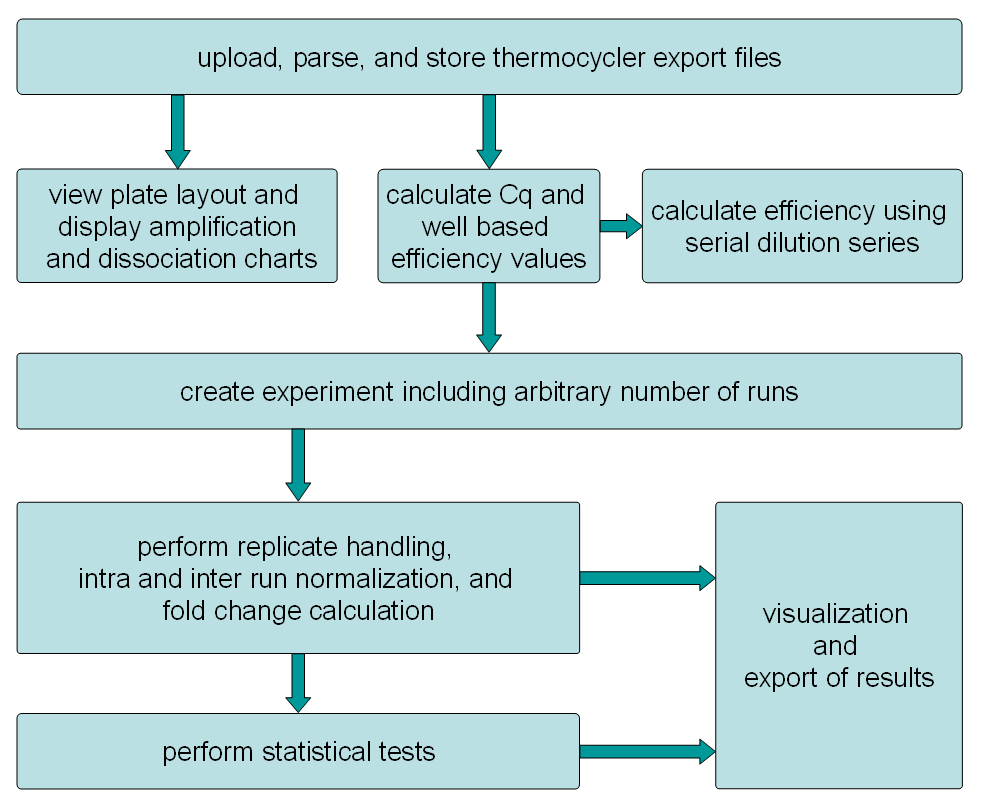
**Analysis pipeline**. This figure illustrates the analysis pipeline implemented in the QPCR application.

QPCR is an application which integrates storage, management, and analysis of qPCR experiments into one single tool. Implemented as a web application it can be accessed by a web browser from every network connected computer and therefore supports the often decentralized work of biologists. It parses files generated by qPCR instruments, stores data and results in a database, and performs analyses on the imported data. Moreover, it allows conducting of statistical tests and provides several ways to visualize and export the calculated results (Figure 1).

### Parsing files and calculation of Cq/efficiency values

Data files are uploaded into the application using a single file upload dialog or an integrated Java applet which supports uploading of multiple files at once. An upload zone lists all available files and allows querying and downloading of data previously uploaded. All files are stored in a user defined directory facilitating the backup of project critical files.

After uploading the exported files into the QPCR application, a list of all files which have not yet been processed is shown. The user can select single or multiple files for parsing. Moreover, Cq and amplification efficiency values can be automatically calculated after the files have been parsed using one or several different methods.

During parsing all relevant data is extracted, including plate setup, fluorescence measurements, and qPCR instrument specifications and stored in the database. In contrast to many available analysis tools the application is able to import qPCR data files without the need for additional file manipulations and therefore reduces error-prone and cumbersome manual work. In addition to the already existing data file parsers the application can be easily extended to support other vendors due to the modularity of the platform and the used plug-in mechanism.

Once the data is parsed and stored in the database, Cq and amplification efficiency values are calculated based on the fluorescence measurements. Several published and widely-used algorithms were implemented; two different algorithms to calculate Cq together with efficiency values, three different algorithms to calculate solely the amplification efficiency, and one method to calculate the Cq value are available (see Table 1).

**Table 1:** Algorithms used in the QPCR web application

Name	Description	Author	Cq	Efficiency
Cy0	The method operates on raw fluorescence data and fits a five parameter Richard's curve. Next it calculates the tangent of the inflection point and intersects it with the abscissa axis which results in the Cq value.	Guescini *et al. *[27]	✓	-

LinReg	The method operates on background corrected data and uses log transformed values to construct a slope with the highest correlation to the original curve. The parameters of this slope are used to calculate the amplification efficiency.	Ramakers *et al. *[8]	-	✓

Miner	The method operates on raw fluorescence data and fits a four-parameter logistic curve to determine the Cq value by using the second derivative maximum. The efficiency is calculated by using a weighted average of a fitted exponential curve.	Zhao *et al. *[28]	✓	✓

RutledGene	The method operates on raw fluorescence data and fits a four-parametric sigmoid function to calculate efficiency values.	Rutledge *et al. *[29]	-	✓

SoFar	The method operates on raw fluorescence data and fits exponential or sigmoid function on smoothed data to calculate Cq and efficiency values.	Wilhelm *et al. *[30]	✓	✓

TAQ	The method operates on raw fluorescence data and performs a linear regression on log transformed data to determine the efficiency values.	Ostermeier *et al. *[31]	-	✓

The progress of all active parser or analyzer background tasks is displayed on a view that automatically updates the current status. As soon as a process has finished a message is shown at the top of the page. For each process a log file is created which informs the user about the outcome of the performed job. A color scheme helps to quickly identify the jobs that have not finished successfully.

During parsing of uploaded files a *Run *is created in the application which is a direct representation of the performed qPCR run. It stores information about the hardware, software, thermocycler profile, and category.

Each *Run *contains a plate which consists of multiple wells that store information about the sample, target, passive reference, task, and omitted status. The plate layout can be displayed in a list and each well can be edited to correct inconsistencies or to omit it from further analysis.

**Figure 2:**
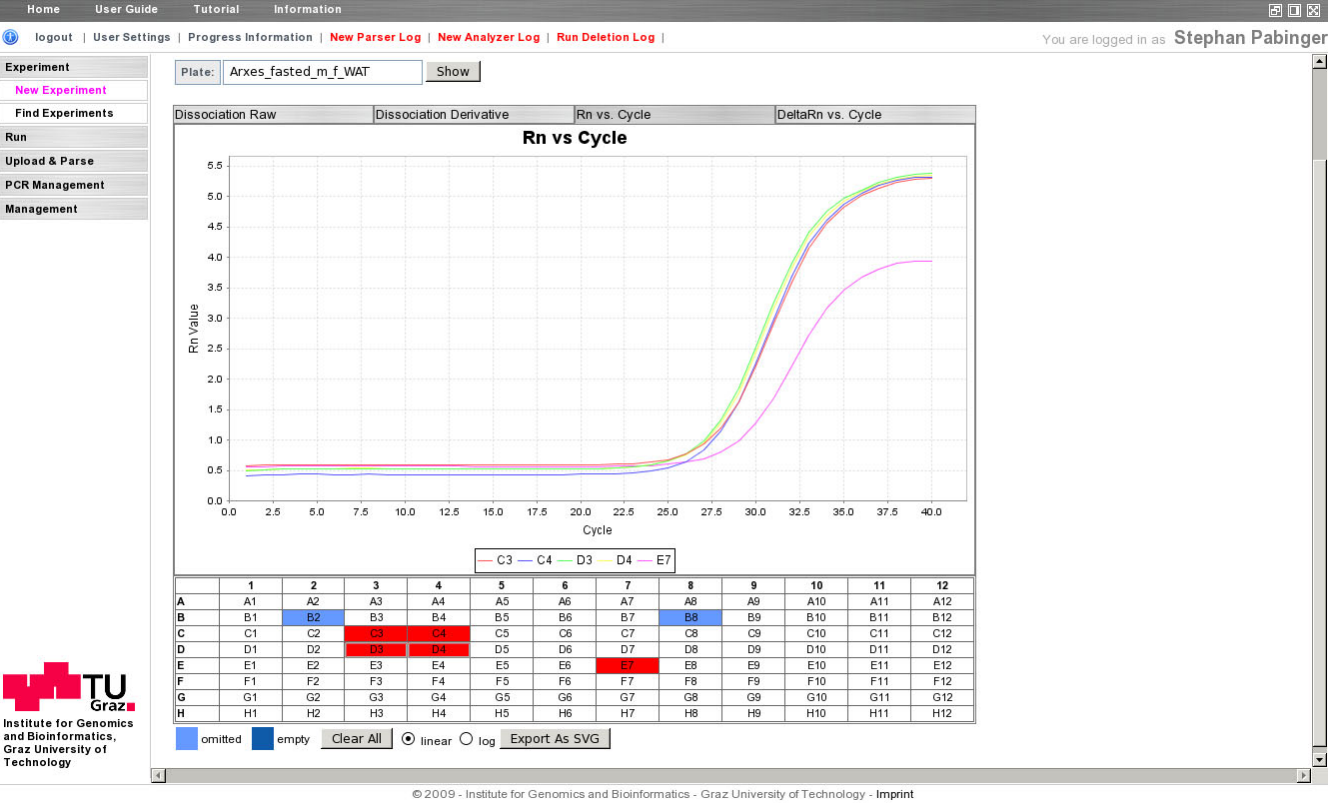
**Graphical representation of the plate layout**. The tabbed bar at the top is used to switch between different chart types. The chart itself features tool tips and provides a legend. Beneath the chart is a representation of the plate layout that is adapted to the plate size (96/384 wells, linear layout). Selected wells are colored in red, omitted and empty wells in blue.

Additionally, QPCR provides a graphical representation of the plate layout by showing a grid which displays sample, target, and status information of each well. By selecting an arbitrary number of wells, charts of amplification (raw and background subtracted) and dissociation (raw and derivative) curves are displayed (Figure 2). This view is helpful to evaluate the performance of the PCR for each well and is useful to perform a quick quality check of the conducted qPCR run.

### Analysis of experiments

After Cq and efficiency values have been determined, experiments consisting of one or multiple runs are subjected to subsequent analysis steps. Several plates can be combined into one experiment. In order to support a flexible and adaptable analysis of experiments, the application allows selecting of specific samples and genes to be used in subsequent analysis steps. Moreover, the Cq calculation method, the efficiency method, and the reference genes can be defined.

Four different ways to consider amplification efficiencies in the analysis have been implemented: (1) setting a single efficiency value for all targets, (2) manually defining the efficiency for each target, (3) using efficiencies derived from dilution series for each target, and (4) using calculated efficiencies for each well. Several different efficiencies values for a target, calculated by serial dilution series, can be stored in the database.

Normalization of experiments is based on a method proposed by Hellemans *et al. *[5] and includes averaging of technical replicates, normalization against reference genes, inter-run calibration, and calculation of quality control parameters. Technical replicates are averaged either within one plate or over all plates of the experiment depending on the analysis setting. In the next step all samples of one gene are referenced to the arithmetic mean Cq value across all samples for this gene. Thereafter the user selected type of efficiency is considered for each target and the samples are normalized to the selected reference genes. If reaction specific efficiency has been selected the efficiency is averaged for each target. Depending on the analysis setting the application supports spreading of reference genes across multiple runs or uses reference genes for each run independently. Finally, inter-run calibrators are automatically detected and are used to normalize results between different qPCR runs.

Quality control parameters for reference genes are calculated based on a method described by Vandesompele *et al. *[4]. When multiple reference genes are selected the coefficient of variation and the gene stability value M are calculated. These parameters are helpful for selecting and evaluating reference genes. Additionally, QPCR performs outlier detection by calculating the difference in quantification cycle value between technical replicates and allows highlighting those that have a larger difference than a user defined threshold. Moreover, quality control checks are performed to test if a no template control (NTC) is present for each target.

Fold change ratios of the calculated normalized Cq values can be calculated by referencing them to one or multiple samples. All analysis setup parameters are automatically stored in the database and are loaded when the experiment is analyzed again. Additionally, each analysis setup can be stored under a user defined name. Throughout the whole analysis process proper error propagation is performed using methods described in [5,23].

**Figure 3:**
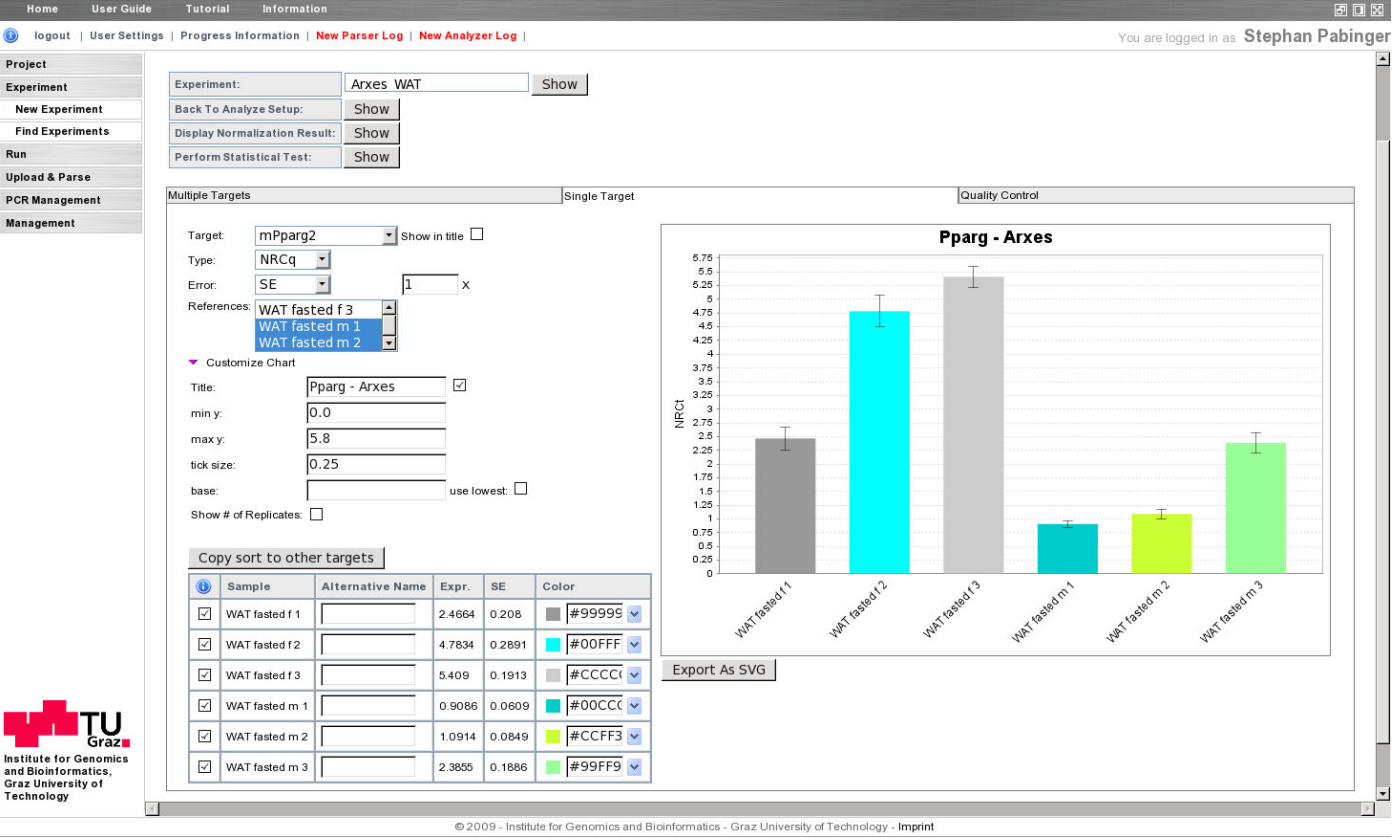
**Visualization of normalized relative quantities**. The tabbed bar is used to switch between views that display multiple targets at once, one target at a time (displayed), or quality control parameters. On the left side the user can define various parameters including the displayed target, the specific result, the presented error, and the reference samples. The list of displayed samples can be reordered using drag and drop, samples may be excluded from the chart, and for each sample an alternative name and an individual color can be assigned.

During the development of the QPCR application special attention was laid on the accurate and user-friendly visualization of calculated results. Therefore, the application allows to display and export results of every important analysis step. The generated figures are highly customizable and are designed to be usable in publications without further manipulation. Among other parameters QPCR allows to define color, labeling, sort sequence, and data type to be used in histogram charts. Cq values normalized by reference genes and calibrators are presented as histograms displaying results of one gene or multiple genes at once (Figure 3). Every result throughout the analysis pipeline can be exported in tab-delimited or spreadsheet format (txt, csv, xls) to be used in external applications.

### Conducting statistical tests

The final step in the analysis pipeline is the comparison of samples using statistical tests (e.g.: biological replicates, samples of a time series). The application allows to group samples into an arbitrary number of classes which are tested for their significant difference against one defined reference class. QPCR includes several statistical tests to compute p-values such as ANOVA, student's t-test, and a permutation based test which makes no assumption on the distribution of the data. Tests can be conducted on either untransformed or log2 transformed values. The application allows adjusting the calculated p-value by supporting several established correction methods for multiple testing [24].

**Figure 4:**
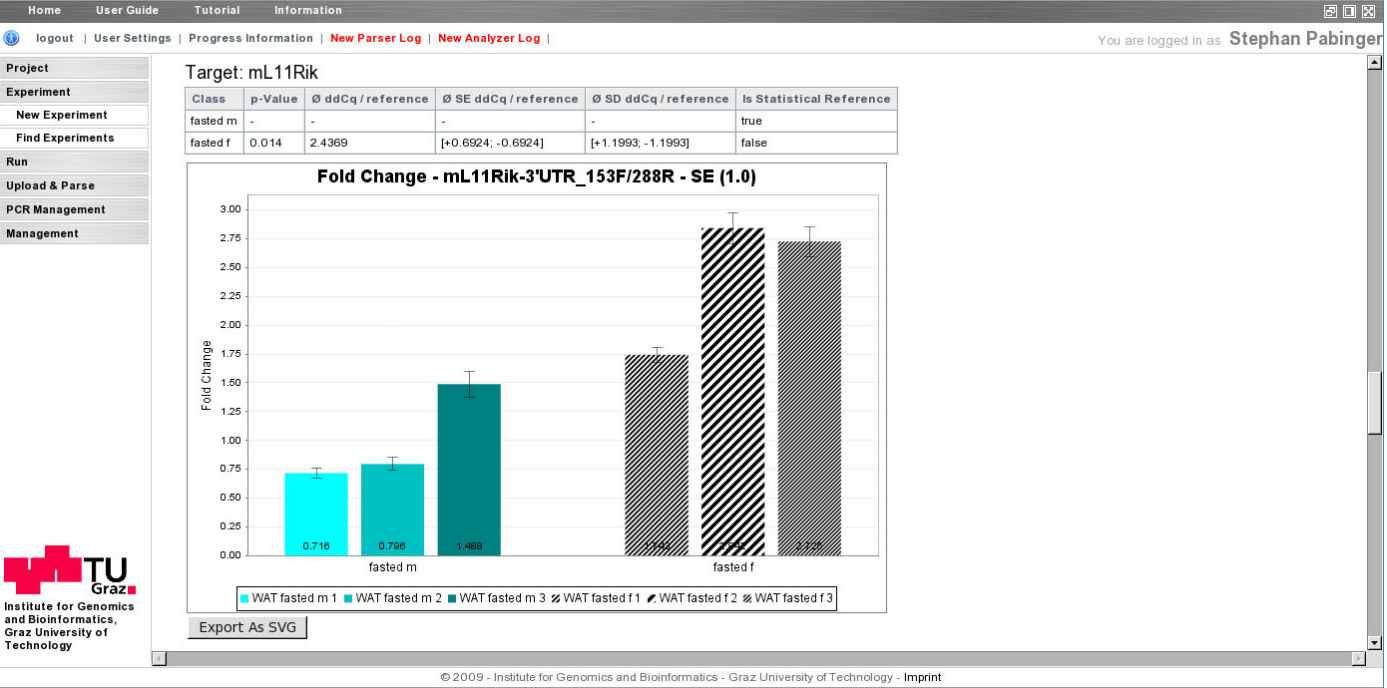
**Visualization of a statistical test result**. The statistical test was used to test two classes of biological replicates for their significant differences, whereas class "*fasted m" *was used as reference class. The table shows the calculated p-Value and parameters. Samples of each class are grouped together and marked in different colors or shapes.

Calculated test results are displayed for each class and can be exported for further analysis. Moreover, the fold changes of samples are displayed in histogram charts in which samples of each class are grouped together. Every class is assigned to a specific user defined color or shape that is used in different shades to group the samples of one class (Figure 4).

### General data entry and query

The application provides views of every entity to (1) manually enter data and (2) list available items. Entry views consist of mandatory and optional fields and use drop down selection lists to specify references to other entities. Entered data is checked for validity and the user is informed about erroneous inputs. List views present the data in tabular form and support paging, sorting, and querying for any combination of the available attributes. Moreover, queries can be stored in the database for later use.

## Discussion

We have developed an integrated platform for the analysis and management of qPCR experiment data using state-of-the-art software technology. The uniqueness of the application is defined by the support of various qPCR instruments, multiple data analyzers, and statistical methods, as well as the coverage of the complete analysis pipeline including proper error propagation. Moreover, it provides a flexible plug-in mechanism to incorporate new parsers and methods and allows generation of highly customizable charts. A comparison of features between QPCR and several other popular qPCR analysis tools is provided in Table 2.

**Table 2:** Comparison of qPCR analysis tools

Feature	QPCR	StatMiner 3.0 (Integromics) [32]	qBase 1.3.5 [5]	qBasePlus 1.1 (Biogazelle) [33]	GenEx 4.4.2 (MultiD) [34]	qPCR-DAMS [6]
Import (Applied Biosystems, Lightcycler, BioRad, Excel)	✓^1^/✓^2^/-/✓	✓/-/-/✓	✓/✓/✓/✓	✓/✓/✓/✓	-/-/-/✓	-/-/-/✓

Absolute/relative quantification	-/✓	✓/✓	-/✓	-/✓	✓/✓	✓/✓

RDML compliant	-^3^	-	-	✓	-	-

Calculate Cq	✓	-	-	-	-	✓

Calculate reaction specific efficiency	✓	-	-	-	-	-

Calculate target specific efficiency	✓	✓	✓	✓	✓	-

Includes geNorm, Normfinder	✓/-	✓/✓	✓/-	✓/-	✓/✓	-/-

Normalization	✓	✓	✓	✓	✓	✓

Inter-run calibration	✓	-	✓	✓	-	✓

Calculate fold change ratios	✓	✓	✓	✓	✓	✓

Quality control	✓	✓	✓	✓	-	✓

Statistical tests	✓	✓	-	-	✓	-

Clustering	-	✓	-	-	✓	-

Correlation	-	-	-	-	✓	-

Error propagation	✓	-	✓	✓	-	-

2D, 3D, Scatter, Bar plots	✓/-/-/✓	✓/-/-/✓	-/-/-/✓	-/-/-/✓	✓/✓/✓/✓	-/-/-/-

Multi-user functionality	✓	-	-	-	-	-

Database storage	✓	✓	-	-	-	✓

Web/Standalone/Plugin	✓/-/-	-/✓/-	-/-/✓^4^	-/✓/-	-/✓/-	-/-/✓^5^

Freely available	✓	-	✓	-	-	✓

(1) ABI 7000, ABI 7500, ABI 7900; (2) LightCycler 2.0, LightCycler 480; (3) Uses the suggested nomenclature; (4) Microsoft Excel based tool: (5) Microsoft Access based tool

The capability to import and parse data without the need for further file manipulations is an integral part of the application which avoids errors during the analysis and reduces the time to analyze the experimental data. As most of the available qPCR software tools rely on special formatted input files it was a prerequisite of the platform to be able to directly parse files generated by the qPCR instruments software suits. Moreover, the system is not confined to a specific manufacturer and can therefore be used in laboratories equipped with qPCR instruments from different vendors.

QPCR includes established and widely used methods for the calculation of Cq and amplification efficiency values and supports an easy integration of new algorithms. This framework does not limit the researcher to one specific approach and allows incorporation of newly developed analysis methods. Furthermore, it is of great value as different experimental situations need to be considered separately and it remains up to individual researches to identify the method most appropriate for their experimental conditions [25]. QPCR allows to store several different analysis settings for each experiment and calculates quality control parameters which help to evaluate the performed analysis. Incorporating several different methods to include the amplification efficiency enhances the flexibility of the application and allows adapting the analysis to the experimental conditions or laboratory practices. Particularly, supporting the widely used calculation of efficiency based on serial dilution series increases the acceptance in the qPCR community.

An often underestimated drawback of using multiple tools to analyze qPCR experiments is the lack of support for assessment of error propagation. Therefore the final error is often based solely on the standard deviation of biological replicates which can lead to false biological interpretations. The QPCR application addresses this problem and includes assessment of error propagation throughout the whole analysis pipeline covering technical replicate handling, normalization, inter-run calibration, referencing against samples, and biological replicate handling. The implemented method is based on Taylor series expansion which allows direct calculation of the full probability distribution and is in contrast to Monte Carlo based methods computationally inexpensive [26].

Special focus was laid on the presentation of analysis results. QPCR provides an interface which uses state-of-the-art software technologies to generate highly customizable charts that are designed to be ready for publication. Since many available tools do not provide a suitable graphical representation of the calculated results, Microsoft Excel is often used to create figures which require manual import and/or conversion of data. QPCR combines the calculation and presentation of results into one single tool which reduces analysis time and avoids additional potential error-prone steps. A flowchart displaying each analysis step and its suggested method is included into the user guide.

The recent developments of data exchange formats (RDML) and guidelines describing the minimum information about qPCR experiments (MIQE) could become an important part in standardizing qPCR experimental data. QPCR already integrates the suggested nomenclature and RDML support will be implemented as soon as the relevant Java libraries are available. Once established in the qPCR community these initiatives will allow a standardized exchange of data between software tools and facilitate the comparison of qPCR experiments.

Using three-tier software architecture that separates the presentation, the business, and the database layer enables not only easy maintenance but also allows distribution of the computing load to several servers. As more and more data needs to be analyzed this design may be very valuable in the future.

The use of a database allows easy querying and comparing of data and guarantees data integrity. The implemented plug-in framework, which is used for including data file parsers, analysis methods, and statistical algorithms, ensures that the application is adaptable to new developments and allows the effortless integration of innovative scientific methods.

## Conclusion

We have developed QPCR, a system for the storage, management, and analysis of qPCR data. It integrates the complete analysis workflow, ranging from Cq determination over normalization and statistical analysis to visualization, into a single application. The analysis time is significantly reduced and complex analyses can now be compared within a single or across multiple laboratories. Optimal usability has been ensured by involving biologists throughout the entire development process and by extensive tests in a laboratory setting. Given the incorporation of several analysis methods and the flexibility due to the use of standard software technology and plug-in mechanism, the developed application could be of great interest to the qPCR community.

## Availability and requirements

• Project name: QPCR

• Project home page: 

• Operating system: Solaris, Linux, Windows, Mac OS X

• Programming language: Java

• Other requirements: Java JDK 1.6.x, Oracle™ 9i or PostgreSQL™ 8.0.x, a server with at least 1 GB of main memory (2 GB are recommended) available to the application

• License: IGB-TUG Software License

• Any restrictions to use by non-academics: IGB-TUG Software License

Installation of the application is provided through an installer and should be completed within one hour provided the necessary database access rights are granted. We recommend installing the application on a central server by a system administrator. Step-by-step instructions are provided at the projects web site together with the installer file. The reference installation of QPCR is running on a SUN Fire™ X4600 M2 6 × dual core Opteron server (Sun Microsystems Ges.m.b.H, Vienna, Austria) with 24 GB of memory running Solaris and using a dedicated Oracle 10 g database server. Attached is a Storage Area Network (EVA 5000, Hewlett-Packard Ges.m.b.H., Vienna, Austria) with 9.5 TBytes net capacity.

## Authors' contributions

SP designed the application and drafted the manuscript. He was responsible for implementation of the database, the development the data presentation and many parts of the business logic. GGT contributed to conception and design of the application and helped drafting the manuscript. RS improved the data file parsers and analysis methods. HE gave valuable input regarding the usability of the platform. RR participated in the design and implementation of the application and helped drafting the manuscript. ZT was responsible for the overall project coordination. All authors gave final approval of the version to be published.

## References

[B1] Wong ML, Medrano JF (2005). Real-time PCR for mRNA quantitation. Biotechniques.

[B2] Livak KJ, Schmittgen TD (2001). Analysis of relative gene expression data using real-time quantitative PCR and the 2(-Delta Delta C(T)) Method. Methods.

[B3] Pfaffl MW (2001). A new mathematical model for relative quantification in real-time RT-PCR. Nucleic Acids Res.

[B4] Vandesompele J, De P, Pattyn F, Poppe B, Van R, De P, Speleman F (2002). Accurate normalization of real-time quantitative RT-PCR data by geometric averaging of multiple internal control genes. Genome Biol.

[B5] Hellemans J, Mortier GR, De P, Speleman F, Vandesompele J (2007). qBase relative quantification framework and software for management and automated analysis of real-time quantitative PCR data. Genome Biol.

[B6] Jin N, He K, Liu L (2006). qPCR-DAMS: a database tool to analyze, manage, and store both relative and absolute quantitative real-time PCR data. Physiol Genomics.

[B7] Simon P (2003). Q-Gene: processing quantitative real-time RT-PCR data. Bioinformatics.

[B8] Ramakers C, Ruijter JM, Deprez RH, Moorman AF (2003). Assumption-free analysis of quantitative real-time polymerase chain reaction (PCR) data. Neurosci Lett.

[B9] Bustin SA (2002). Quantification of mRNA using real-time reverse transcription PCR (RT-PCR): trends and problems. J Mol Endocrinol.

[B10] Bustin SA, Benes V, Garson JA, Hellemans J, Huggett J, Kubista M, Mueller R, Nolan T, Pfaffl MW, Shipley GL, Vandesompele J, Wittwer CT (2009). The MIQE Guidelines: Minimum Information for Publication of Quantitative Real-Time PCR Experiments. Clin Chem.

[B11] Lefever S, Hellemans J, Pattyn F, Przybylski DR, Taylor C, Geurts R, Untergasser A, Vandesompele J (2009). RDML: structured language and reporting guidelines for real-time quantitative PCR data. Nucleic Acids Res.

[B12] Gosling J, Joy B, Steele G, Bracha G (2005). The Java(TM) Language Specification.

[B13] JBoss Group (2008). JBoss Application Server. http://www.jboss.org/jbossas/.

[B14] Apache Software Foundation (2006). Apache Struts. http://struts.apache.org/.

[B15] Getahead (2008). DWR: Easy AJAX for JAVA. http://directwebremoting.org.

[B16] Prototype Core Team (2009). Prototype: JavaScript Framework. http://www.prototypejs.org/.

[B17] John Resig and jQuery Team (2009). jQuery. http://jquery.com/.

[B18] Gilbert David (2008). The JFreeChart Class Library. http://www.jfree.org/jfreechart/.

[B19] Wittwer CT, Ririe KM, Andrew RV, David DA, Gundry RA, Balis UJ (1997). The LightCycler: a microvolume multisample fluorimeter with rapid temperature control. Biotechniques.

[B20] Booch G, Rumbaugh J, Jacobson I (2005). The Unified Modeling Language User Guide.

[B21] AndroMDA Core Team (2007). AndroMDA. http://www.andromda.org/.

[B22] Maurer M, Molidor R, Sturn A, Hartler J, Hackl H, Stocker G, Prokesch A, Scheideler M, Trajanoski Z (2005). MARS: microarray analysis, retrieval, and storage system. BMC Bioinformatics.

[B23] Larionov A, Krause A, Miller W (2005). A standard curve based method for relative real time PCR data processing. BMC Bioinformatics.

[B24] Dudoit S, Shaffer JP, Boldrick J (2002). Multiple Hypothesis Testing in Microarray Experiments. U C Berkeley Division of Biostatistics Working Paper Series Working Paper 110.

[B25] Bustin SA, Benes V, Nolan T, Pfaffl MW (2005). Quantitative real-time RT-PCR – a perspective. J Mol Endocrinol.

[B26] Gerards BM (1998). Error Propagation In Environmental Modelling With GIS.

[B27] Guescini M, Sisti D, Rocchi MB, Stocchi L, Stocchi V (2008). A new real-time PCR method to overcome significant quantitative inaccuracy due to slight amplification inhibition. BMC Bioinformatics.

[B28] Zhao S, Fernald RD (2005). Comprehensive algorithm for quantitative real-time polymerase chain reaction. J Comput Biol.

[B29] Rutledge RG (2004). Sigmoidal curve-fitting redefines quantitative real-time PCR with the prospective of developing automated high-throughput applications. Nucleic Acids Res.

[B30] Wilhelm J, Pingoud A, Hahn M (2003). SoFAR: software for fully automatic evaluation of real-time PCR data. Biotechniques.

[B31] Ostermeier GC, Liu Z, Martins RP, Bharadwaj RR, Ellis J, Draghici S, Krawetz SA (2003). Nuclear matrix association of the human beta-globin locus utilizing a novel approach to quantitative real-time PCR. Nucleic Acids Res.

[B32] Integromics (2009). RealTime StatMiner. http://www.integromics.com/StatMiner.php.

[B33] Biogazelle (2009). qBasePlus. http://www.biogazelle.com/site/products/qbaseplus.

[B34] MultiD (2009). GenEx. http://www.multid.se/genex.html.

